# Longitudinal Assessment of Neurocognitive Outcomes and Correlation with Limbic System Radiation Doses in Patients Undergoing Radiotherapy for Central Nervous System Tumours: A preliminary report

**DOI:** 10.12688/f1000research.166763.1

**Published:** 2025-09-10

**Authors:** Prerana Baruah, Athiyamaan MS, Sourjya Banerjee, Johan Sunny, Paul Simon, Muralidhar Pai, Mayur Kamath, Arjun Shetty, Challapalli Srinivas, Dilson Lobo, Sahana Srinivasan, Pooja Prakash, Abinesh E, Abhishek Krishna

**Affiliations:** 1 Department of Radiation Oncology, Kasturba Medical College Mangalore, Manipal Academy of Higher Education, Karnataka, Manipal, India; 2 Department of Neurosurgery, Kasturba Medical College Mangalore, Manipal Academy of Higher Education, Karnataka, Manipal, India

**Keywords:** Cognitive Dysfunction, Brain Neoplasms, Radiotherapy, Cognition, Glioma

## Abstract

**Background:**

Radiation therapy (RT) for central nervous system (CNS) malignancies can result in neurocognitive decline, affecting quality of life. While hippocampal-sparing approaches are well documented, limited data exist on the impact of radiation dose to other cognitive structures. This prospective study evaluates domain-specific cognitive changes following RT and explores correlations with radiation dose to key brain regions.

**Methodology:**

Twenty-five patients with primary CNS tumours undergoing focal radiotherapy, with or without concurrent chemotherapy, were enrolled. Neurocognitive function was assessed using the Addenbrooke’s Cognitive Examination III (ACE-III) tool, pre- and six months post-treatment. Radiation dose-volume histogram (DVH) data were analyzed for structures including the hippocampus, amygdala, cerebellum, and corpus callosum. Patients were stratified based on ACE-III score decline (≥3 points vs. <3 points), and dose-response correlations were examined.

**Results:**

A statistically significant decline in mean ACE-III scores was observed post-treatment (pre: 89.48 ± 6.84 vs. post: 87.91 ± 6.29, p = 0.007). Attention, language, and fluency were the most affected domains. Patients with cognitive decline received higher radiation doses to all examined structures, with the corpus callosum showing the strongest association (mean dose in decline group: 35.91 Gy vs. 30.70 Gy; p = 0.440). However, no dose-response relationship reached statistical significance.

**Conclusion:**

This study highlights early, domain-specific neurocognitive decline following focal RT for CNS tumours, with attention and language being most vulnerable. Corpus callosum dose emerged as a key correlate. These findings suggest the need to broaden neuroprotection strategies beyond the hippocampus and emphasize the value of incorporating routine neurocognitive assessment.

## Introduction

The prevalence of cranial nervous system tumours has increased due to improved diagnosis, treatment protocols, and multimodal treatment approaches. According to GLOBOCAN 2022 data, the global incidence of Central Nervous System (CNS) tumours is approximately 322,000. ^1^ As per National Cancer Registry Program (NCRP), the Indian data records the incidence of CNS tumours ranging from 5 to 10 cases per 100,000 population, accounting for approximately 2% of all malignancies. ^2^

The management of gliomas presents a unique challenge, not only due to their aggressive biological behaviour but also because of their profound effects on neurocognitive function. As advancements in neurosurgical techniques, chemotherapy, and radiotherapy have improved survival rates, the focus has increasingly shifted toward preserving the quality of life, particularly cognitive function. Neurocognitive impairment is a common finding in glioma patients, impacting functions such as memory, executive function, attention, and language function ^3, 4^ While surgical resection, chemotherapy, and radiotherapy are the mainstay modalities of treatment, the effect of these modalities on neurocognitive function is a significant area of research. Specifically, radiation therapy has been associated with cognitive impairment, most probably because of radiation-induced damage to critical cognitive and limbic system structures of the brain such as the hippocampus, corpus callosum, and amygdala ^5, 6^ New radiation techniques such as volumetric-modulated arc therapy (VMAT) have been developed to better localize the tumour with less exposure to normal neural tissue, potentially reducing neurocognitive toxic effects. ^7^

The neurocognitive effects of radiation are multifactorial, involving direct damage to neurons, vascular damage, and neuroinflammation. The extent of cognitive impairment depends on factors like tumour location, radiation dose, fractionation schedules, and patient-specific vulnerability. ^6, 8^ Previous studies have highlighted the importance of assessing cognitive outcomes following radiotherapy using standardized neurocognitive tests. ^9, 10^ The Addenbrooke’s Cognitive Assessment (ACA) Scale is one such validated instrument for assessing various cognitive domains, including memory, attention, fluency, language, and visuospatial processing, and is a valuable instrument for glioma patients undergoing radiotherapy. ^11^

Several studies have previously considered neurocognitive outcomes after surgery and radiotherapy for glioma patients. ^6, 12^ Talacchi et al. described that tumour resection by itself can be associated with cognitive change, irrespective of adjuvant treatments. Although the influence of the dose of radiation on the remaining limbic structures and the cerebellum is underrepresented in the literature, hippocampal dose constraint studies have proved that lowering the dose of radiation to the hippocampus can spare cognition, reaffirming the position of accurate radiation planning. ^5^

The purpose of this study was to examine the correlation of dose delivered to the critical limbic system structures—hypothalamus, corpus callosum, amygdala—and anterior and posterior cerebellum, and resulting neurocognitive change measured by the ACE Scale.

## Methodology

This prospective observational study was conducted to assess early neurocognitive changes in patients with primary central nervous system (CNS) tumours receiving radiation therapy and to evaluate the correlation between cognitive outcomes and radiation dose to specific brain substructures involved in cognition. The study received approval from the institutional ethics committee, and written informed consent was obtained from all participants prior to start of the study.

Adult patients (≥18 years) with newly diagnosed, histologically confirmed gliomas of WHO Grade II to IV were included. Eligibility criteria required a Karnofsky Performance Score (KPS) of seventy or above. Patients were excluded if they had prior cranial radiotherapy, pre-existing cognitive impairment, neurodegenerative disorders, psychiatric illness affecting cognition, history of traumatic brain injury, or other neurological conditions known to affect cognitive function. Recurrent or residual tumours from prior treatments were also excluded.

Demographic and clinical data were collected at baseline, including age, sex, socioeconomic indicators. Tumour-related data included anatomical location, laterality, WHO grade, histological subtype, and molecular markers. Extent of surgical resection was categorized as biopsy, subtotal resection, or gross total resection.

### Cognitive assessment protocol

Neurocognitive function was assessed using the Addenbrooke’s Cognitive Examination III (ACE-III), which evaluates five domains: attention/orientation (18 points), memory (26 points), verbal fluency (14 points), language (26 points), and visuospatial abilities (16 points), with a total score range of 0–100. ^11^ Cognitive assessments were performed prior to radiation therapy, at six months, 1 year and 2 years post radiation therapy. This preliminary analysis reports findings from the baseline and 6-month assessments. All assessments were conducted in person by trained clinical staff in the outpatient setting. Tests were administered in the participant’s preferred language. Where needed, trained interpreters were involved to facilitate communication. Each domain score and the total score were recorded. A total ACE-III scores above 87 was categorized as normal cognition, 83–87 as inconclusive, and below 83 as impaired.

### Treatment details

Patients were planned for external beam radiation therapy using intensity modulated radiotherapy (IMRT) or volumetric-modulated arc therapy (VMAT). Surgical intervention included either biopsy or maximum safe resection, based on the tumour location and feasibility. Radiotherapy was administered in the adjuvant setting for all patients.

Radiation was delivered to a dose of 54 to 60 Gy in 1.8–2.0 Gy fractions depending on tumour characteristics and patient clinical status. Treatment planning included immobilization with thermoplastic masks and acquisition of planning computed tomography (CT) images with 3 mm slice thickness. Neurocognitive assessments were done post-surgery and prior to radiation therapy in all patients, and these were considered as the post-surgical baseline values.

Planning Magnetic resonance imaging (MRI) sequences were co-registered with the CT for accurate target and organ-at-risk (OAR) delineation. The gross tumour volume (GTV), clinical target volume (CTV), and planning target volume (PTV) were defined according to Radiation Therapy Oncology Group (RTOG) and the European Organization for Research and Treatment of Cancer (EORTC) guidelines. ^13, 14^ In addition to standard OARs, specific neurocognitive structures were contoured, including the bilateral hippocampi, corpus callosum, amygdalae, and anterior and posterior cerebellum using European Particle Therapy Network (EPTN) guidelines. ^15^ Treatment plans were generated and reviewed by a team consisting of radiation oncologists and medical physicists. Radiation dose distributions were recorded for subsequent analysis. Chemotherapy was administered based on tumour histology and standard clinical guidelines. Patients with glioblastoma or anaplastic gliomas received concurrent temozolomide (75 mg/m ^2^/day) during radiotherapy, followed by adjuvant temozolomide in 28-day cycles. Patients with 1p/19q codeleted oligodendrogliomas received procarbazine, lomustine, and vincristine (PCV) chemotherapy as indicated. If patients did not tolerate PCV, Temozolomide was administered. Chemotherapy data, including regimen and number of cycles, were documented for each participant.

Dose-volume histogram (DVH) data were used to extract radiation dose metrics for the hippocampi, corpus callosum, amygdalae, anterior cerebellum, and posterior cerebellum. For each structure, the minimum (Dmin), maximum (Dmax), and mean (Dmean) radiation doses were recorded. In cases where only one hippocampus was spared due to tumour involvement on one side, only the contralateral side was used for analysis. These values were collected and organized for groupwise comparison. Participants were stratified into two groups based on change in total ACE-III score between baseline and follow-up. Group A included patients with a decline of ≥3 points, while Group B included patients with a decline of <3 points or no change. The threshold of three points was selected based on literature indicating that such a change is clinically relevant in ACE-III performance.

### Statistical analysis

All statistical analyses were conducted using IBM SPSS Statistics software (version 27). Descriptive statistics were used to summarize demographic and clinical characteristics. Continuous variables were expressed as mean and standard deviation (SD) for normally distributed data, and as median and interquartile range (IQR) for non-normally distributed data. Categorical variables were reported as absolute frequencies and percentages. Assess whether the distribution of continuous variables followed a normal distribution, the Shapiro–Wilk test was applied. Based on the outcome of the normality test, appropriate parametric or non-parametric tests were selected for subsequent comparisons. The primary outcome variable was the change in total Addenbrooke’s Cognitive Examination III (ACE-III) score from post-surgery baseline to six months post-radiotherapy. Paired sample t-tests were used to compare post-surgery and post-radiation ACE-III total and domain-specific scores for normally distributed data. For non-normally distributed score data, the Wilcoxon signed-rank test was used as a non-parametric alternative. For subgroup analysis, patients were stratified into two groups based on change in total ACE-III score - Group A: Patients with a decline of ≥3 points and Group B: Patients with a decline of <3 points or no change. Between-group, comparisons of radiation dose metrics to each of the contoured brain substructures were conducted using independent samples t-tests for normally distributed data and the Mann–Whitney U test for non-normally distributed data.

To evaluate associations between radiation dose and change in cognitive scores as continuous variables, correlation analyses were performed using Pearson’s correlation coefficient for normally distributed variables and Spearman’s rank-order correlation for non-normally distributed variables. Correlation strength was interpreted using standard thresholds (e.g., 0.1–0.3 = weak, 0.3–0.5 = moderate, >0.5 = strong).

For all hypothesis testing, a two-sided p-value <0.05 was considered statistically significant. Where appropriate, 95% confidence intervals (CIs) were calculated and reported to quantify the precision of effect size estimates. Data completeness was monitored throughout the study. Cases with missing post-treatment ACE-III data (due to death, dropout, or loss to follow-up) were excluded from paired statistical analyses but included in baseline descriptive summaries. No imputation was performed for missing values.

## Results

### Patient characteristics

A total of twenty-five patients with histologically confirmed primary central nervous system tumours were included in the study. The median age at diagnosis was 42 years. The initial symptoms varied, though headache emerged as the most reported complaint, affecting ten patients (40%) followed by limb weakness (36%), giddiness (12%), seizures (8%), and altered sensorium (8%. Comorbidities were present in eight patients (32%). Among these, diabetes mellitus and deep vein thrombosis were most frequently observed.

In terms of histopathology, astrocytomas were the most diagnosed tumours, found in eleven patients (44%). Glioblastomas were seen in eight cases (32%), while six patients (24%) had oligodendrogliomas. The frontal lobe was the most frequently involved anatomical site (24%), followed by the temporal lobe (16%), parietal region (12%), frontoparietal area (12%), and the insular cortex (12%). Multifocal disease was observed in one patient presenting with a tumour in the cerebellopontine angle. Grading according to the WHO classification showed that 44% (n = 11) of tumours were Grade 4, 32% (n = 8) were Grade 2, and 24% (n = 6) were Grade 3. IDH mutation analysis was available for fifteen patients; of these, 7 (28%) tested positive for the mutation, while 3 (12%) were classified as wild type. Radiation dose delivered ranged from 54 Gy to 60 Gy. The details of the patient characteristics are outlined in Table 1.

**Table 1.** Patient characteristics.

Characteristic	Detail	n (%)
Age (years)	Median	42
Gender	Male	17 (68%)
	Female	8 (32%)
Presenting Complaint	Headache	10 (40%)
	Motor weakness	9 (36%)
	Giddiness	3 (12%)
	Seizures	2 (8%)
	Altered sensorium	2 (8%)
Histology	Astrocytoma	11 (44%)
	Glioblastoma	8 (32%)
	Oligodendroglioma	6 (24%)
Tumour Grade	Grade II	8 (32%)
	Grade III	6 (24%)
	Grade IV	11 (44%)
Radiation Dose	54 Gy	8 (32%)
	60 Gy	17 (68%)
Temozolomide	Concurrent & Adjuvant	20 (80%)
	No	5 (20%)

### Neurocognitive assessments

The ACE-III assessed five cognitive domains: memory, attention, verbal fluency, language, and visuospatial abilities. The mean post-surgery baseline ACE-III total score was 89.48 ± 6.48 (median: 92; 86–94). Based on pre-defined thresholds, 72% (n = 18) of patients demonstrated normal cognition (score >87), 12% (n = 3) had inconclusive scores (83–87), and 16% (n = 4) scored in the impaired range (<83). At six months, the mean post-treatment ACE-III total score was 87.91 ± 6.29 (median: 89; 85–92, *p* = 0.57). Following treatment, 60% (n = 15) had scores in the normal range, 4% (n = 1) in the inconclusive range, and 16% (n = 4) in the impaired range.

A ≥3-point decline in total ACE-III score was observed in 68% (n = 17) of patients, while 24% (n = 6) showed no meaningful change, and 8% (n = 2) demonstrated improvement. Domain-wise comparisons revealed a statistically significant reduction in attention scores post-treatment (mean: 14.45 ± 2.04 vs 15.16 ± 2.03; *p* = 0.026). Reductions in language (23.91 ± 1.90 vs 24.20 ± 1.73; *p* = 0.090) and fluency (12.73 ± 1.70 vs 12.84 ± 1.52; *p* = 0.104) approached significance. Changes in memory (23.23 ± 1.80 vs 23.56 ± 1.78; *p* = 0.184) and visuospatial abilities (13.68 ± 1.32 vs 13.80 ± 1.61; *p* = 0.134) were not statistically significant ( Table 2).

**Table 2.** Neurocognitive assessment - Pre (baseline) and 6 months post radiation.

Domains	N	Timing	Mean	SD	Median	IQR		P value
Attention [0/18]	25	Pre-Radiation	15.16	2.03	15	14	17	0.026
		Post Radiation	14.45	2.04	15	14	16	
Memory [0/26]	25	Pre-Radiation	23.56	1.78	24	22	25	0.184
		Post Radiation	23.23	1.80	24	22	25	
Fluency [0/14]	25	Pre-Radiation	12.84	1.52	13	13	14	0.104
		Post Radiation	12.73	1.70	13	13	14	
Language [0/26]	25	Pre-Radiation	24.20	1.73	25	24	25	0.090
		Post Radiation	23.91	1.90	25	23	25	
Visuospatial abilities [0/16]	25	Pre-Radiation	13.80	1.61	14	14	15	0.134
		Post Radiation	13.68	1.32	14	13	15	
Total Score	25	Pre-Radiation	89.48	6.84	92	86	94	0.57
		Post Radiation	87.91	6.29	89	85	92	

Radiation dose to cognitive structures

Radiation dose-volume histogram (DVH) data were analyzed for all patients. Among the delineated cognitive-related brain structures, the corpus callosum received the highest mean radiation dose (mean: 34.96 ± 14.56 Gy; median: 38.71 Gy; range: 2.22–59.73 Gy). The amygdala and hippocampus received mean doses of 21.23 ± 13.43 Gy (median: 21.09 Gy; range: 0.84–44.05 Gy) and 19.86 ± 12.20 Gy (median: 17.20 Gy; range: 1.72–50.25 Gy), respectively. The anterior cerebellum received a mean dose of 13.60 ± 12.49 Gy (median: 10.63 Gy; range: 0.50–42.45 Gy), while the posterior cerebellum received the lowest mean dose (9.30 ± 10.59 Gy; median: 5.18 Gy; range: 0.35–37.24 Gy). In the subgroup of 13 patients with bilateral hippocampal contouring (unilateral tumour involvement), the mean hippocampal dose was higher, recorded at 34.07 ± 13.82 Gy (median: 35.87 Gy; range: 4.03–56.38 Gy). The maximum doses in this subgroup reached 63.14 Gy for the hippocampus, 61.33 Gy for the amygdala, and 65.15 Gy for the corpus callosum ( Table 3).

**Table 3.** Radiation dose to structures involved in cognition.

Structure	Dose parameter	Mean	SD	Median	IQR		Range	
					Percentile 25	Percentile 75	Minimum	Maximum
Hippocampus	D Min	10.13	9.44	7.84	4.46	12.79	0.67	37.57
	D Max	32.33	17.29	29.17	21.47	42.53	0.26	63.13
	D Mean	19.86	12.20	17.20	12.58	25.18	1.72	50.24
Amygdala	D Min	14.60	10.80	11.37	6.46	18.39	0.64	38.53
	D Max	29.47	18.70	25.82	17.86	44.12	1.13	61.32
	D Mean	21.23	13.43	21.09	12.57	31.73	0.83	44.04
Anterior Cerebellum	D Min	3.02	3.97	1.45	0.92	2.52	0.21	17.23
	D Max	35.99	21.44	42.04	16.63	55.07	1.72	63.89
	D Mean	13.60	12.49	10.63	4.24	19.86	0.50	42.44
Posterior Cerebellum	D Min	2.23	2.76	1.31	0.75	2.18	0.16	12.72
	D Max	31.33	24.23	24.22	12.71	55.35	0.74	64.89
	D Mean	9.30	10.59	5.18	2.11	13.56	0.34	37.24
Corpus Callosum	D Min	15.09	12.55	10.79	7.42	19.90	1.04	44.36
	D Max	52.29	11.62	55.34	46.77	60.75	12.27	65.14
	D Mean	34.96	14.56	38.71	23.88	44.34	2.21	59.72

Range of dose for the structures (hippocampus, corpus callosum, anterior cerebellum, posterior cerebellum, amygdala).

### Dose–response analysis and cognitive correlation

Patients were stratified into two cohorts based on the degree of change in ACE-III score following treatment: Group A (≥3-point decline, n = 15) and Group B (<3-point decline, n = 10). Group A exhibited numerically higher radiation dose exposure across all analyzed brain structures. The hippocampal mean dose was 20.29 ± 16.71 Gy in Group A and 19.86 ± 8.94 Gy in Group B ( *p* = 0.940). Corresponding maximum hippocampal doses were 33.38 ± 20.59 Gy and 30.38 ± 15.58 Gy, respectively ( *p* = 0.707). The mean doses to amygdala were 22.23 ± 17.42 Gy in Group A versus 22.10 ± 11.88 Gy in Group B ( *p* = 0.984). Anterior cerebellar mean doses were 14.78 ± 15.69 Gy in Group A and 15.20 ± 11.51 Gy in Group B ( *p* = 0.944), while posterior cerebellar doses were 10.73 ± 12.67 Gy and 10.26 ± 10.51 Gy, respectively ( *p* = 0.928).

The corpus callosum exhibited the largest absolute inter-group difference, with a mean dose of 35.91 ± 17.45 Gy in Group A compared to 30.70 ± 12.92 Gy in Group B ( *p* = 0.440). Minimum and maximum dose comparisons in the corpus callosum also followed a similar trend, though not statistically significant. In patients with bilateral hippocampi, the mean dose was 15.54 ± 23.80 Gy in Group A and 21.89 ± 17.31 Gy in Group B ( *p* = 0.486). No statistically significant differences were observed in any of the analyzed dose parameters between the two groups ( Table 4).

**Table 4.** Comparison of radiation dose parameters to brain structures between patients with and without significant neurocognitive decline.

Structure	Dose parameter	Group A			Group B			
		Mean	SD	Median	Mean	SD	Median	P value
Hippocampus	D Min	11.16	12.45	5.27	10.26	8.66	8.67	0.847
	D Max	33.38	20.59	22.72	30.38	15.58	28.77	0.707
	D Mean	20.29	16.71	14.46	19.86	8.94	17.64	0.940
Amygdala	D Min	15.66	13.46	14.19	15.17	10.44	13.19	0.927
	D Max	30.38	24.32	25.93	30.57	15.89	26.62	0.982
	D Mean	22.23	17.42	21.26	22.10	11.88	21.39	0.984
Anterior Cerebellum	D Min	3.25	3.78	1.56	3.37	4.77	1.33	0.955
	D Max	31.88	27.13	18.05	41.36	16.17	43.42	0.329
	D Mean	14.78	15.69	7.67	15.20	11.51	11.35	0.944
Posterior Cerebellum	D Min	2.29	2.35	1.40	2.56	3.47	1.20	0.841
	D Max	29.66	29.21	13.03	36.06	19.72	42.37	0.556
	D Mean	10.73	12.67	5.18	10.26	10.51	6.23	0.928
Corpus Callosum	D Min	15.63	15.02	7.49	13.30	9.58	11.29	0.669
	D Max	54.95	9.03	56.65	47.50	13.28	49.67	0.164
	D Mean	35.91	17.45	33.66	30.70	12.92	33.92	0.440
Hippocampus Bilateral Cases	D Min	8.38	15.40	3.24	10.56	10.65	8.67	0.705
	D Max	20.27	30.49	16.66	32.41	22.48	42.69	0.305
	D Mean	15.54	23.80	10.28	21.89	17.31	28.59	0.486

## Discussion

Neurocognitive function has become an essential parameter in the management of primary CNS malignancies, especially as survival improves with multimodal treatments. Cognitive impairment in these patients arises not only from the tumour itself but also from interventions such as surgery, chemotherapy, and radiation therapy. This prospective study aimed to evaluate neurocognitive decline following focal radiotherapy (RT) in glioma patients using the Addenbrooke’s Cognitive Examination III (ACE-III), and to explore associations between cognitive change and radiation dose to key neuroanatomical structures. A statistically significant decline in total ACE-III scores was observed post-radiotherapy, with mean scores decreasing from 89.48 ± 6.84 to 87.91 ± 6.29 (p = 0.007). Domain-wise, attention, language, and fluency were the most affected, while memory and visuospatial abilities were preserved. Notably, patients who exhibited greater cognitive decline tended to have received higher doses to key neural structures, particularly the corpus callosum.

The ACE-III tool enabled domain-specific assessment of cognitive change, capturing subtle yet meaningful declines in attention, language, and fluency. Compared to tools like the MMSE (Mini-Mental State Examination) and HVLT-R (Hopkins Verbal Learning Test-Revised), the ACE-III offers broader coverage and greater sensitivity to early changes. ^11^ This allowed for more precise mapping of cognitive changes to anatomical structures.

These findings align with the growing understanding that radiotherapy to limbic system structures can lead to early cognitive impairment when critical neural structures are exposed to moderate-to-high doses. ^16^ While hippocampal avoidance has been the focus of most cognitive-sparing radiotherapy studies, including the NRG-CC001 trial which demonstrated memory preservation with hippocampal-sparing WBRT plus memantine, our data highlight that attention and fluency were more prominently affected domains. ^17^ This discrepancy may reflect the focal, rather than whole-brain, nature of RT in glioma patients.

The corpus callosum, a major interhemispheric white matter tract responsible for integrating cortical functions and sustaining attention, showed a strong trend toward correlation with cognitive decline in our cohort. Although statistical significance was not reached across all metrics, higher doses (>35 Gy) to the corpus callosum were associated with greater reductions in ACE-III scores. This is supported by the study by Redmond et al., who demonstrated that radiation exposure to white matter tracts such as the corpus callosum and superior longitudinal fasciculus predicted neurocognitive dysfunction in glioma patients. ^18^ Our findings are further corroborated by Chapman et al., who used diffusion tensor imaging to show microstructural degeneration of the corpus callosum associated with attention and processing speed deficits. ^19^

Of the 25 patients, 7 (28%) had abnormal ACE-III scores at baseline post-surgery. In contrast, 8 (32%) had abnormal scores at 6 months post-radiotherapy. This corresponds to a 4% increase in cognitive dysfunction after radiotherapy. The 28% baseline impairment reflects tumour- and surgery-related cognitive effects. Hence it may not be justified to refer radiotherapy as the primary cause for the observed cognitive decline, and therefore the results should be interpreted keeping in mind other possible clinical factors and outcomes.

Although the hippocampus remains central to memory preservation strategies, our study did not find a statistically significant relationship between hippocampal dose and post-treatment memory decline. This contrasts with results from Gondi et al., who reported hippocampal dose-dependent memory loss, particularly in low-grade or benign intracranial tumours. ^20^ The absence of a similar association in our study may be attributed to lower hippocampal doses overall, contralateral compensation in unilateral lesions, or sample size constraints. Our study also examined radiation exposure to other critical brain structures. The amygdala and cerebellum, although not primary targets, received variable radiation doses depending on tumour location. Although no significant correlation with cognitive outcomes was noted, prior studies suggest these structures contribute to cognitive-affective integration and executive function. ^21^ A few Studies in literature have similarly identified radiation dose to frontal and temporal lobes as predictors of decline in language and fluency. ^22, 23^ The findings also resonate with Haisraely et al., who proposed expanding dose-sparing protocols beyond the hippocampus to include white matter tracts. ^24^ Our results support this direction, showing that cognitive impairment involves a distributed neural network rather than isolated memory pathways alone. Talacchi et al. reported that cognitive diminution post-surgery can occur in up to 30–40% of patients, and more likely in executive functions, memory, and attention, domains largely mediated by frontal-subcortical networks. ^4^ As our study population had higher number of frontal lobe lesions, the cognitive impairment that we observed may reflect the impact of surgery on cognition.

Despite the consistency with literature, our study has few limitations. The current analysis includes a smaller cohort and a short follow up time, which may have obscured significant dose-response associations. However, patient recruitment is ongoing, and future analyses with a larger sample size and extended follow-up are expected to strengthen the findings. Functional neuroimaging techniques, such as volumetric MRI or diffusion tensor imaging, were not incorporated at this stage, which may have further elucidated the structural basis of observed cognitive changes. However, patient recruitment is ongoing, and future analyses with a larger sample size and extended follow-up are expected to strengthen the findings and allow for more robust dose-volume-outcome correlations.

Future studies should include larger, multicentric cohorts with longitudinal cognitive assessment and advanced neuroimaging. Expanding cognitive-sparing strategies to include midline white matter tracts, cerebellar structures, and language circuits may improve outcomes.

## Conclusion

This study demonstrates that early neurocognitive decline following focal RT exists, particularly affecting attention and language. While hippocampal dose remains relevant, the corpus callosum appears to play a critical role in early cognitive impairment. These findings underscore the need for comprehensive neuroanatomical preservation in radiotherapy planning.

## Consent

Written informed consent to take part in the research has been obtained from all patients when indicated. Consent for publication is not applicable as all data is anonymized, and no images are being published.

## Ethics statement

The Institutional Ethics Committee of Kasturba Medical College approved this study, Mangalore, vide protocol no. IEC KMC MLR 09/2024/554 dated 19.09.2024. The study was conducted in accordance with the ethical guidelines and regulations set forth by the institution. The study has been conducted according to the principles expressed in the Declaration of Helsinki.

## Author contributions

All authors contributed equally to the conceptualization, methodology, data collection, analysis, manuscript writing, and revision. All authors have read and approved the final version of the manuscript.

## Data Availability

Data Repository Used: FIGSHARE Link: https://doi.org/10.6084/m9.figshare.29245502.v1 Citation: Krishna A. Neurocognitive Study Datasheet. figshare; 2025 [cited 2025 Jul 1]. https://figshare.com/articles/dataset/Neurocognitive_Study_Datasheet/29245502/1 ^25^ This project contains the following underlying data:
•
DATA_Neuro_Figshare.xlsx Data are available under the terms of the CC by 4.0 license ( https://creativecommons.org/licenses/by/4.0/)
