# Supplementary material for: Longitudinal Assessment of Neurocognitive Outcomes and Correlation with Limbic System Radiation Doses in Patients Undergoing Radiotherapy for Central Nervous System Tumours: A preliminary report
Source: F1000Res. 2025 Sep 10;14:895. [Version 1] doi: 10.12688/f1000research.166763.1 (PMC12744206; doi:10.12688/f1000research.166763.1)
Supplement: Peer review history

# Reviewer report: Reviewer response for version 1

**DOI:** 10.5256/f1000research.183800.r429724

**Published:** 2025-12-27

**Authors:** Seema Nagpal

**Affiliations:** 1 Stanford Cancer Institute, Stanford, USA

Kudos to the authors for presenting a well written and well designed study of cognitive changes seen in patients with glioma who are treated with radiation. This is important work reflecting "on the ground truths" that many of is in the field accept, but are challenging to prove definitively, as this study demonstrates. There are trends, across the board, towards worse outcomes with more/higher exposure, but not quite meeting statistical significance.

 Those of us who see these patients every day know that they are impaired and larger fields seem to be worse, so why can't we demonstrate it? The ACEIII appears to be a better test than some others, but was also designed for patients with AD or, less iatrogenic dementias. I don't have an alternate battery to propose, but simply to make clear that none of these batteries were designed to capture the types of cognitive injury patterns we might see in these patients. Perhaps a few more sentences about that?

 Otherwise, I congratulate the authors on a study that was likely quite a bit of work...and working toward the common goal of making our patient's lives better by using our tools smarter.

Is the work clearly and accurately presented and does it cite the current literature?

Yes

If applicable, is the statistical analysis and its interpretation appropriate?

Yes

Are all the source data underlying the results available to ensure full reproducibility?

Yes

Is the study design appropriate and is the work technically sound?

Yes

Are the conclusions drawn adequately supported by the results?

Partly

Are sufficient details of methods and analysis provided to allow replication by others?

Yes

Reviewer Expertise:

Glioblastoma, glioma, brain metastases, leptomeningeal metastases (and radiation sparing!)

I confirm that I have read this submission and believe that I have an appropriate level of expertise to confirm that it is of an acceptable scientific standard.

# Reviewer report: Reviewer response for version 1

**DOI:** 10.5256/f1000research.183800.r429715

**Published:** 2025-12-27

**Authors:** Dr. Sandesh B Rao

**Affiliations:** 1 Department of Radiation Oncology, Father Muller Medical College,, Mangalore, India

It is a well conducted study assessing the neurocognitive outcomes and correlation with limbic system radiation doses in patients undergoing radiation for brain tumors. Literature review, study design, methodology and results appear logical. Interpretation of the result with statistical analysis looks logical.Conclusions are also make sense. In total a good study which can be considered for publication.

Is the work clearly and accurately presented and does it cite the current literature?

Yes

If applicable, is the statistical analysis and its interpretation appropriate?

I cannot comment. A qualified statistician is required.

Are all the source data underlying the results available to ensure full reproducibility?

Yes

Is the study design appropriate and is the work technically sound?

Yes

Are the conclusions drawn adequately supported by the results?

Yes

Are sufficient details of methods and analysis provided to allow replication by others?

Yes

Reviewer Expertise:

Head and neck Oncology, Breast oncology, Gynac oncology and CNS Oncology

I confirm that I have read this submission and believe that I have an appropriate level of expertise to confirm that it is of an acceptable scientific standard.

# Reviewer report: Reviewer response for version 1

**DOI:** 10.5256/f1000research.183800.r429723

**Published:** 2025-11-12

**Authors:** Lanisha Sequeira

**Affiliations:** 1 Father Muller Medical College, Mangalore, India

This is a very good and seldom researched topic. I congratulate the researchers for taking up the research. The study is well conducted and i have only few minor comments.

 Introduction:

 Change “cranial nervous system tumours” to “central nervous system (CNS) tumours” for terminological consistency.

 Methodlogy:

 Correct grammar — should read “To assess whether the distribution of continuous variables followed a normal distribution, the Shapiro–Wilk test was applied.

 Results: Table 1 -Altered sensorium (8%.” → Missing closing parenthesis.

 Discussion. Write a note on use of temozolomide or concurrent PCV in the patients and its impact on the neurocognition.

Is the work clearly and accurately presented and does it cite the current literature?

Yes

If applicable, is the statistical analysis and its interpretation appropriate?

Yes

Are all the source data underlying the results available to ensure full reproducibility?

Yes

Is the study design appropriate and is the work technically sound?

Yes

Are the conclusions drawn adequately supported by the results?

Yes

Are sufficient details of methods and analysis provided to allow replication by others?

Yes

Reviewer Expertise:

Oncology

I confirm that I have read this submission and believe that I have an appropriate level of expertise to confirm that it is of an acceptable scientific standard.
